# Role of indoleamine 2, 3-dioxygenase 1 in immunosuppression of breast cancer

**DOI:** 10.1016/j.cpt.2023.11.001

**Published:** 2023-11-07

**Authors:** Pratyasha Sarangi

**Affiliations:** School of Biotechnology, Kalinga Institute of Industrial Technology, Bhubaneswar, Odisha 751024, India

**Keywords:** IDO1, Breast cancer, IDO1 inhibitor, Immunosuppression, Tumor microenvironment

## Abstract

Breast cancer (BC) contributes greatly to global cancer incidence and is the main cause of cancer-related deaths among women globally. It is a complex disease characterized by numerous subtypes with distinct clinical manifestations. Immune checkpoint inhibitors (ICIs) are not effective in all patients and have been associated with tumor resistance and immunosuppression. Because amino acid (AA)-catabolizing enzymes have been shown to regulate immunosuppressive effects, this review investigated the immunosuppressive roles of indoleamine 2,3-dioxygenase 1 (IDO1), a tryptophan (Trp)-catabolizing enzyme, which is overexpressed in various metastatic tumors. It promotes immunomodulatory effects by depleting Trp in the regional microenvironment. This leads to a reduction in the number of immunogenic immune cells, such as effector T and natural killer (NK) cells, and an increase in tolerogenic immune cells, such as regulatory T (Treg) cells. The BC tumor microenvironment (TME) establishes a supportive niche where cancer cells can interact with immune cells and neighboring endothelial cells and is thus a feasible target for cancer therapy. In many immunological contexts, IDO1 regulates immune control by causing regional metabolic changes in the TME and tissue environment, which may further affect the maturation of systemic immunological tolerance. In the development of effective treatment targets and approaches, it is essential to understand the immunomodulatory effects exerted by AA-catabolizing enzymes, such as IDO1, on the components of the TME.

## Highlights

• Immune checkpoint inhibitors (ICIs) show limited efficacy and have been linked to immunomodulation and immune escape.
• Amino acid (AA)-catabolizing enzymes can cause immunomodulation and immune escape in various cancers.
• Breast cancer (BC) is a complex condition characterized by several subtypes with distinct clinical manifestations.
• Indoleamine 2,3-dioxygenase 1(IDO1) causes tryptophan (Trp) depletion and interacts with immune cells to promote immune escape.
• Many IDO1 inhibitor trials are currently ongoing, but other enzymes of the IDO pathway impede their success.

## Graphical abstract

**Figure:**
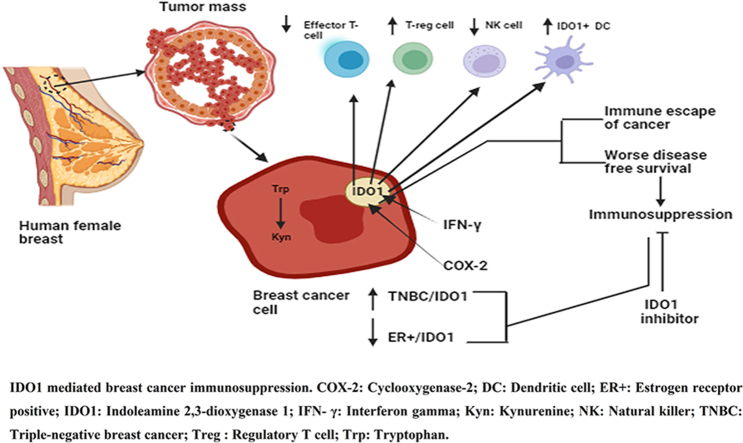

## Introduction

The immune system protects the body from diseases, infections, foreign substances, or damaged cells through a series of reactions. In 1856, Paul Ehrlich presented initial evidence that the immune system can recognize antigens presented by cancer cells that are foreign to the body and ultimately eliminate them. The theory of immunotolerance was later proposed and suggested that tumors evade or bypass T cell recognition and function to prevent attacks by the immune system. Immunotolerance, also known as immune escape, is the propensity of malignant cells to evade immune system defenses. Because they influence the development and application of anticancer and adjuvant therapies, the molecular mechanisms that promote immune tolerance during neoplasm genesis and progression have been the focus of considerable research. Immune checkpoint inhibitors (ICIs) are central to cancer immunotherapy and the primary treatment choice for advanced-stage cancer. The interaction between cancer and immune cells changes as the tumor progresses through different stages of neoplastic development, which allows tumors to evade the immune system. During cancer immunoediting—a process consisting of elimination, equilibrium, and escape^1^—intricate interactions occur between the immune system and tumor cells within the tumor microenvironment (TME) that not only influence the antigenicity of the tumor but also shape its immunogenicity.^1^

Breast cancer (BC) is typically not regarded as highly immunogenic. However, clinical trials have successfully implemented strategies that utilize the immune system, particularly against triple-negative BC (TNBC).^2^ Although ICI therapy shows promise against metastatic cancers, including BC, its effectiveness is limited to a small number of patients.^2^ This limited clinical efficacy may be attributed to a low tumor mutational burden, which leads to the production of fewer immunogenic neoantigens and a highly immunosuppressive TME.^2^ Traditional cancer immunotherapy is frequently challenged by limited efficacy, owing to diverse mechanisms that promote resistance and immune evasion. The relatively low response rates in patients with BC suggest the participation of various mechanisms of immune evasion. Over the last few decades, various enzymes involved in amino acid (AA) metabolism have been associated with the suppression of immune responses against tumors,^3^ either by depleting AAs from the TME or producing metabolites with immunosuppressive effects.^3^ These enzymes are categorized according to the AA upon which they act. Inducible nitric oxide synthase (iNOS) is the sole form of NOS generated by immune cells. Arg1 and Arg2 (type 1 and 2 arginase, respectively), break down arginine, a semi-essential AA. Interleukin 4 (IL-4)-induced gene 1 (*IL4I1*) breaks down phenylalanine to produce phenylpyruvate, hydrogen peroxide (H_2_O_2_), and ammonia (NH_3_). Tryptophan (Trp), an essential AA, is metabolized by Trp 2,3-dioxygenase (TDO) and type 1 and 2 indoleamine 2,3-dioxygenases (IDO1 and IDO2).

IDO plays a vital role in cancer immunotherapy by catalyzing the synthesis of kynurenine (Kyn) from Trp. Dysregulation of the IDO1 pathway has been implicated in BC. The aim of this review was to investigate the mechanisms that are currently considered to participate in IDO1-mediated immune suppression and its impact on cancer growth. It also provides a comprehensive overview of the association between IDO1 and BC along with the outcomes of clinical trials targeting the IDO1 pathway.

## Immunosuppressive tumor microenvironment of breast cancer

**Figure 1:**
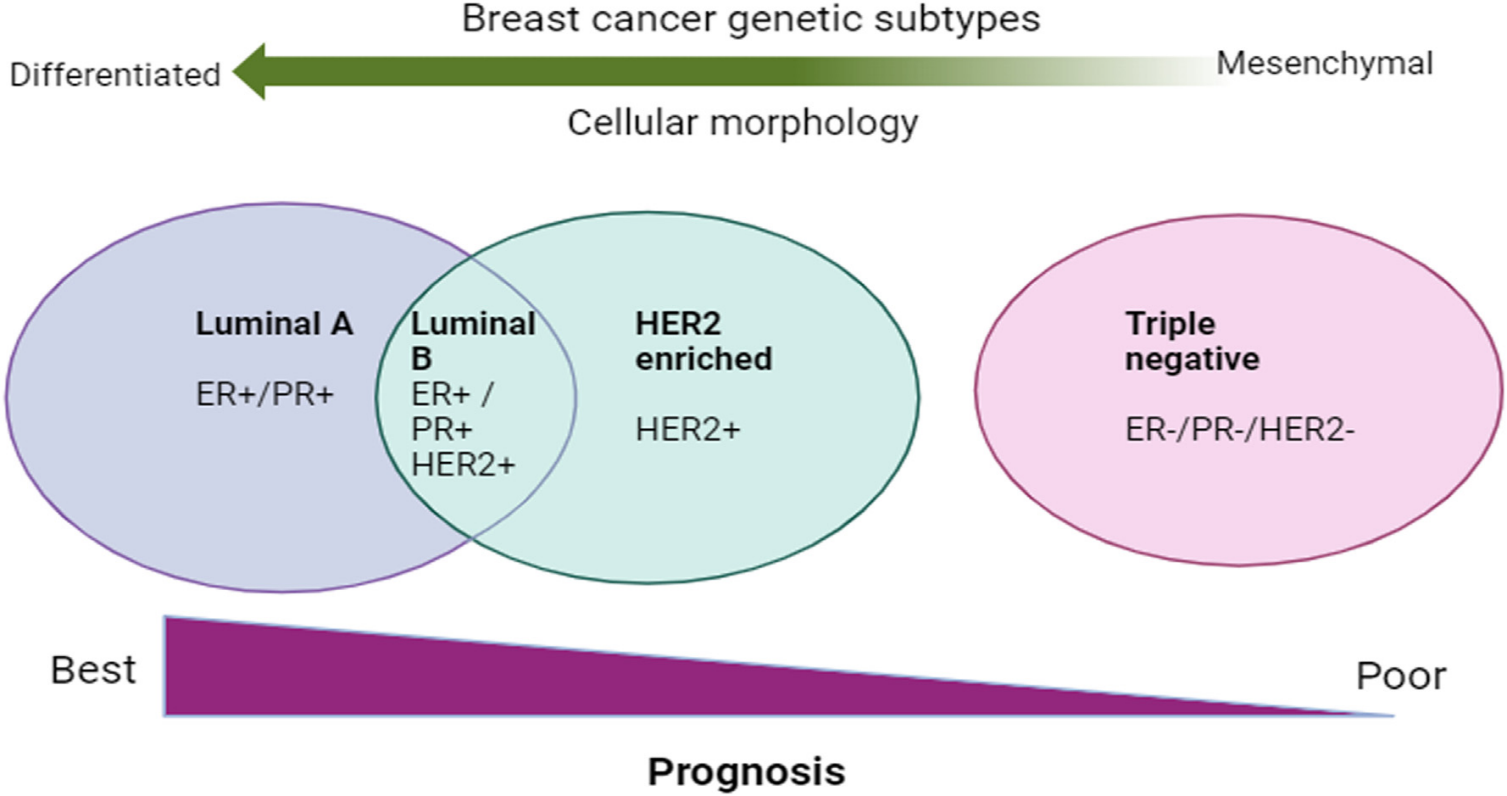
Pictorial representation of BC subtypes. BC: Breast cancer; ER−: Estrogen receptor negative; ER+: Estrogen receptor positive; HER2: Human epidermal growth factor receptor 2; HER2−: Human epidermal growth factor receptor 2 negative; HER2+: Human epidermal growth factor receptor 2 positive; PR−: Progesterone receptor negative; PR+: Progesterone receptor positive.

BC is a diverse condition that encompasses various subtypes, each characterized by distinct biological characteristics and clinical manifestations. BC is the primary contributor to global cancer diagnoses, accounting for 2.6 million cases in 2020 alone, which is approximately 11.7% of all reported cancer cases.^4^ It is the fourth major cause of cancer-related fatalities, resulting in 685,000 deaths in 2020, equivalent to approximately 6.9% of all cancer-related deaths.^4^ BC treatment typically relies on the molecular characteristics of the tumor subtype, which includes luminal-A, luminal-B, human epidermal growth factor receptor 2 (HER2)-enriched, and TNBC [Figure 1]. Among these, luminal-A and -B^5^ have the highest 5-year survival rates, followed by HER2-enriched^6^ and TNBC^7^ [Figure 1]. Molecular categorization of the different BC subtypes has enabled the development of more precise targeted therapies, among which drug-based treatments play a crucial role.

It is also important to examine the relationship between the development of breast tumors and their tolerance to the immune system. A notable recent advancement in this area was the identification of the TME functions facilitating immune evasion. The peritumoral and intratumoral stroma of breast neoplasms are home to a variety of molecular components, which contribute to the complexity of the TME.^8^ Communication between malignant cells and the surrounding microenvironment, which plays a vital role in tumor progression, occurs through an “efferent” mechanism, where cancer cells trigger a response in the stroma, and “afferent” mechanism, where activated stromal cells and modified extracellular matrix (ECM) affect the aggressiveness of cancer cells.^9^^,^^10^ These interactions involve an intricate network of stromal cells, ECM compartments, growth factors, cytokines, enzymes, and hormones. Additionally, the TME occasionally triggers the conversion of epithelial cells into BC stem cells (BCSC).^11^ These cells interact in the TME and stimulate several biological processes that support the development, invasion, angiogenesis, and metastasis of cancer.

### Mammary cancer-associated fibroblasts

Emerging evidence has demonstrated a correlation between mammary cancer-associated fibroblasts (mCAFs) and decreased survival rates among patients with a more aggressive BC tumor phenotype. When breast tumors infiltrate the blood or lymphatic vessels, clusters of mCAFs are formed to shield cancer cells from immune responses, enhance their ability to withstand fluid mechanical forces, decrease programmed cell death, and facilitate invasion of blood vessels. In BC, IL-6 releases tumor necrosis factor (TNF)-α to activate the expression of KDM2A in normal fibroblasts of the breast, transforming them into mCAFs.^12^ This process is facilitated by CD73+ γδ regulatory T (Treg) cells, which create a positive feedback loop with IL-6 via adenosine release. Adenosine then signals CAFs through the adenosine A2B receptor (*A2BR)*/p38 mitogen-activated protein kinase (p38 MAPK) pathway, further enhancing IL-6 secretion.^12^ The presence of CD73+ γδ Tregs also hampers the propensity of CD8+ T cells to effectively eliminate malignant cells, leading to a poorer prognosis.^12^ The interaction between CD73+ γδ Tregs and CAFs, involving IL6-adenosine loops, appears to play an important role in the advancement of BC and suppression of the immune system. Timperi et al. proposed that lipid-associated macrophages (LAMs), recruited through the chemokine (C-X-C motif) ligand (CXCL)12-chemokine (C-X-C-motif) receptor (CXCR4) axis by CAFs, contribute immunosuppression through pro-tumorigenic activities.^13^

### Cancer-associated adipocytes

Breast tissue contains important adipocytes. Cancer-associated adipocytes (CAAs) are prominent components of the BC TME. CAAs originate during the dedifferentiation of adjacent adipocytes in BC.^14^ CAAs contrast standard adipocytes in terms of metabolic activity, adipokine expression, and size. They undergo several biochemical and morphological changes, increasing the production of different adipokines and cytokines, which subsequently foster the growth, development, and proliferation of breast neoplasms.^14^ This is achieved by altering the ECM, modulating aromatase expression, reprogramming the metabolic cycle, and shaping TME immunology. Leptin, an important adipokine, is abundantly expressed by mature adipocytes in breast tissues. Increased serum levels of leptin have been linked to a higher risk, invasiveness, and unfavorable prognosis of BC,^15^ which could be explained by several potential mechanisms. Leptin triggers the activation of the cyclic adenosine monophosphate (cAMP) response element (CRE) and Janus kinase (JAK)/signal transducer and activator of transcription (STAT)3 signaling cascades, leading to the upregulation of cyclin D1. It inhibits the expression of p21, a protein that inhibits the activity of cyclin-dependent kinases (CDKs), consequently decreasing the number of BC cells in the G0/G1 phase and increasing the number of cells in the S phase. These effects promote cell proliferation and regulate apoptosis.^16^ Leptin has also been found to promote focal adhesion kinase (FAK) activation *in vitro*, which weakens adhesion among cells and increases the production of matrix metalloproteinases (MMP)-2 and −9 for the transformation of the ECM and propagation of TNBC cell lines. In contrast, the expression of adiponectin, which inhibits the development and invasion of BC cells, was reduced in CAAs. Adiponectin can selectively bind to the two receptors AdipoR1 and AdipoR2, which inhibits the growth and migration of BC cells. Activation of AMP-activated protein kinase (AMPK) inhibits this mechanism by suppressing the c-Src/mitogen-activated protein kinase (MAPK) and phosphoinositide 3-kinase (PI3K)/AKT/mammalian target of rapamycin (mTOR) signaling cascades. Adiponectin reduces leptin signaling by mitigating STAT3 induction, AKT phosphorylation, and WNT signaling. This is achieved by increasing the expression of the suppressor of cytokine signaling 2 (SOCS2), which efficiently suppresses the development and metastasis of breast neoplasms. Furthermore, adiponectin impedes leptin signaling by reducing the levels of first apoptosis signal (FAS)-related enzymes and sterol regulatory element-binding protein 1 (SREBP-1), thereby inhibiting fatty acid synthesis in BC cells.^17^ BC cells cultured with adipocytes showed increased expression of IL-6, which regulates resistance to drugs, cell survival, and suppression of immune responses by activation of STAT3 and phosphorylation of JAK.

### FoxP3+ regulatory T cells

The majority of tumor-infiltrating lymphocytes (TILs) are composed of T lymphocytes (CD3+).^18^ CD4+, Tregs, and CD8+ cells are the major types of T lymphocytes. The most important antitumor T cells are CD8+ and CD4+. Tregs perform crucial functions in immunosuppression and are characterized by the expression of the transcription factor forkhead box P3 (Foxp3).^19^ Foxp3+ Tregs are the primary TILs in BC. The presence of Foxp3+ Tregs within the immunosuppressive regional microenvironment hampers the efficacy of checkpoint inhibitors in eliciting an antitumor immune response.^20^ The pro-tumor function of Foxp3+ Tregs involves several mechanisms: First, the inhibition of the Notch pathway.^21^ Second, direct suppression occurs when cells come into direct contact, and indirect suppression occurs with the secretion of anti-inflammatory agents, including ILs (IL-4, IL-10).^22^ Third, stimulation of the STAT1/STAT3 signaling cascade and inhibition of cytokine IL-17 and interferon-gamma (IFN-γ) activity.^23^ Jamiyan et al. identified the presence of Foxp3+ Tregs in the stroma of 107 TNBC samples by immunohistochemistry (IHC) and revealed a strong association between low levels of stromal Foxp3+ Tregs and recurrence-free survival (RFS) and overall survival (OS).^24^ Based on IHC, the abundance of Foxp3+/CD25+ TILs and expression of Foxp3+ were positively linked to OS in 43 TNBC cases.^25^ In BC, an elevated number of CD20-expressing B and Treg tumor cells is a promising prognostic indicator. Ge et al. demonstrated that the activation of Tregs specific to tumor antigens in the bone marrow resulted in the accretion of Tregs within the malignant breast tissues. This contributes to both antitumor immune responses and the regional suppression of immune responses within the BC microenvironment.^26^ Further comprehensive research is necessary to determine the prognostic importance of Tregs in BC.

### M2 tumor-associated macrophages

Macrophages are frequently observed in the TME of patients with breast malignancies, where they play a vital role in neoplasm development, resistance to medications, and immunosuppression.^27^ Normally (M1) and alternatively activated (M2) macrophages are two phenotypes of tumor-associated macrophages (TAMs) that exhibit tremendous adaptability.^27^ M2 TAMs are stimulated by reactive oxygen species (ROS) and are immunosuppressive. Their stimulation culminates in the amplification of programmed death ligand 1 (PD-L1) expression via the induction of nuclear factor kappa B (NF-κB) signaling. Additionally, these macrophages release chemokines with immunosuppressive properties, including insulin-like growth factor-binding protein 3 (IGFBP-3), IL-17, IL-1β, IL-4, IL-10, and CXCL1.^28^ M2 TAMs are the most common type of breast tumors.^29^ The primary mechanism involves suppressing the immune responses of antitumor T cells by releasing anti-inflammatory cytokines. IL-10 produced by M2 TAMs controlled the stimulation and proliferation of CD8+ T cells in a mouse model of BC^30^ and inhibited the secretion of IL-12 by dendritic cells (DCs), thereby limiting the CD8+ T cell response.^30^ This effect was validated in that the removal of M2 TAMs from the mouse model restored the antitumor activity of CD8+ T cells.^30^ In addition to cytokine activities, TAMs can inhibit T cell activity by altering l-arginine metabolism^31^ through the secretion of Arg1, an enzyme that breaks down l-arginine into l-ornithine and urea. The depletion of l-arginine directly hampers T cell activity. The elevation of Arg1 expression has been observed in TAMs from an early-stage mammary tumor mouse model and in circulating myeloid cells of patients with BC.^31^ TAMs also enhance the expression of iNOS, which further metabolizes l-arginine.^31^ Notably, TAMs located in the hypoxic region of murine mammary tumors have been reported to restrict cellular responses through the actions of Arg1 and iNOS.^31^ M2 TAMs contribute to immune evasion in BC through the recruitment of immunosuppressive leukocytes. M2 TAMs attract immunosuppressive cells, such as inflammatory monocytes, to the tumor area through chemokine (C–C-motif) ligand (CCL22)/chemokine (C–C-motif) receptor (CCR2) and colony-stimulating factor (CSF)1/CSF 1 receptor (CSF1R) signaling. Another mechanism by which M2 TAMs promote immune evasion is through the inhibition of tumoricidal functions. This loss of the original macrophage functions, such as the ability to kill tumor cells and produce proinflammatory signals, can become a major barrier in controlling tumor growth through the immune system.

### Myeloid-derived suppressor cells

Myeloid-derived suppressor cells (MDSCs) play a crucial function in the immunomodulation network. Monocytic (M)-MDSCs and granulocytic (G)-MDSCs are distinct categories of cells found in circulation. Following treatment with doxorubicin and cyclophosphamide, a notable increase and decrease were observed in the levels of G-MDSCs and M-MDSCs, respectively. Compared with other subtypes of BC, an increased number of MDSCs has been observed in TNBC.^32^ MDSCs are recruited to the primary cancer and metastatic sites through the activation of chemokines CCL22 and CXCL2, dependent on the presence of ΔNp63.^32^ Restriction of glycolysis decreases the MDSC population by inhibiting the expression of granulocyte-macrophage CSF (GM-CSF) and granulocyte (G)-CSF.^33^ Conversely, hypoxia promotes the augmentation of MDSCs and induces the upregulation of PD-L1 expression in the hypoxic microenvironment of 4T1 mice with breast carcinomas.^34^ The 4T1 TNBC model demonstrated the efficient accumulation of immunosuppressive MDSCs by releasing inflammatory cytokines, which in turn creates a permissive pro-metastatic TME.^35^ Research has suggested that chemokine (C–C-motif) ligand (CCL)-5 plays a major role in Rb1 activation and is linked to the immunomodulatory properties of MDSCs, particularly in the G-MDSC subgroup.

### Plasmacytoid dendritic cells

DCs are mononuclear cells that migrate from bone marrow. They participate in initiating the adaptive immune response by presenting antigens to T lymphocytes.^36^ High numbers of DCs are found in various types of carcinoma tissues, including BC.^37^ There are two distinct subsets of DCs—myeloid DCs (mDCs) and plasmacytoid DCs (pDCs)—that originate from the same source but have different life cycles and phenotypes.^38^ In contrast to pDCs, which secrete type I IFN and have greater tolerogenic qualities along with negative prognostic consequences, mDCs primarily activate immune cells. The increased presence of pDCs in BC tissues may contribute to the upregulation of CXCR4 expression, which is likely mediated by TNF-α-induced NF-κB activation. This ultimately leads to the migration of cancer cells toward the lymph nodes through the CXCR4/stromal-cell derived factor (SDF)-1 chemo–attractive axis.^39^ By engaging with inducible co-stimulator ligand (ICOSL) expressed on pDCs, ICOS can regulate the proliferation of Treg cells and secretion of IL-10 by CD4+ T cells that infiltrate BC lesions.^40^ Furthermore, the presence of ICOS + cells has been associated with unfavorable patient outcomes, indicating that ICOS contributes to the repression of T cell-mediated immune responses instead of promoting antitumor immunity through Th1, Th17, or follicular helper T (TFh) cell responses.^40^ This effect of ICOS may be influenced by immunosuppressive factors in the TME, such as transforming growth factor-β (TGF-β) and IL-10.

## Indoleamine 2,3-dioxygenase 1: immunosuppressive tryptophan dioxygenase

Trp is a particularly scarce AA that is necessary for the synthesis of serotonin, melatonin, vitamin B3, and niacin. Trp metabolism has been linked to tumor development and immune system control.^41^ Enzymes such as TDO and IDO1/IDO2 catalyze the first rate-limiting step by promoting the oxidative degradation of the indole group of Trp. These enzymes are necessary for Trp catabolism in the Kyn pathway (KP). IDO and TDO have gained considerable attention owing to their association with diabetes, inflammatory bowel disease, cancer, and mental disorders.

IDO1, which is highly active toward Trp (Km = 20 M), is a heme-containing monomeric enzyme located on chromosome 8p12. IDO1 is frequently activated in various types of human cancers, including BC, and affects the innate immune system, tumor, and stromal cells.^42^ The presence of IDO1 is often associated with a negative prognosis. Its impact on immunosuppression is complex as it involves the inhibition of NK and CD8+ effector T cells and promotes the activity of MDSCs and CD4+ Treg cells.^43^ In many immunological contexts, IDO participates in immune control through regional metabolic changes in the cancer microenvironment and regional tissue environment, which affect the maturation of systemic immune tolerance. The role of IDO1 as a pivotal interface between inflammatory cytokines reflects its ability for creating an inflammatory environment that promotes tumor growth. IFN-γ plays a well-established role in antitumor immune responses, whereas IL-6 is regarded as a pro-tumorigenic cytokine. Although more recent studies have demonstrated that IDO1 is associated with the induction of IL-6, IFN-γ is a major driver of IDO1 induction. Although extensive research has been conducted on the effects of Trp deprivation, the roles of Kyn and other Trp catabolites are unclear. Platten et al. found that Kyn acts as a native ligand for aryl hydrocarbon receptor (AhR), a transcription factor that performs important functions in proinflammatory activities and discovery is particularly relevant in inflammatory carcinogenesis.^44^

**Figure 2:**
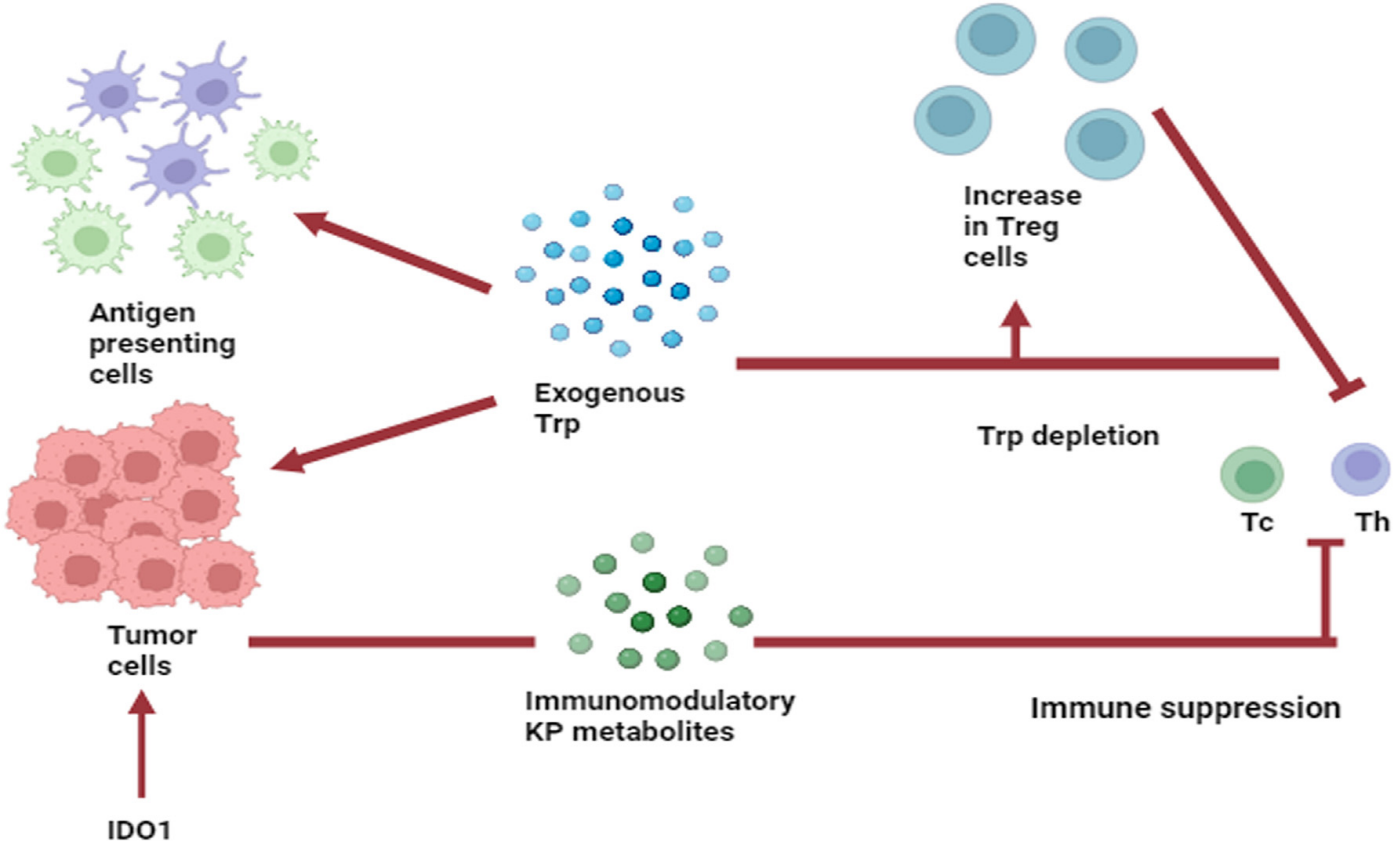
Immune-tolerance mechanism via IDO1 in immunosuppressive cancer microenvironment. The pro-inflammatory cancer environment causes IDO1 overexpression in stomal cells as well as in antigen-presenting cells causing Trp depletion in the surrounding milieu. Due to this, the localized Tc and Th cells become deficient in Trp and stop proliferating due to GCN2 signaling cascade activation. As active T cells become deficient in Trp they undergo Fas-dependent apoptosis and in this manner, CD4+ CD25+FoxP3+ Tregs mature to become the predominant subset of T cells in the surrounding cancer environment causing immunosuppression. IDO1: Indoleamine 2,3-dioxygenase 1; KP: Kynurenine pathway; Tc: Cytotoxic T cell; Th: T helper cell; Treg: Regulatory T cell; Trp: Tryptophan.

In the proinflammatory TME, local Trp levels decrease owing to the upregulation of IDO1 levels in stromal and/or antigen-presenting cells (APCs).^45^ Owing to GCN2 cascade activation, regional T helper (Th) and cytotoxic T (Tc) cells become deficient in Trp, which inhibits their proliferation.^46^ Additionally, active T cells are more susceptible to Fas-dependent apoptosis under Trp deficiency.^46^ Consequently, mature CD4+CD25+Foxp3+ Tregs become the predominant T cell subset in the regional milieu^47^ and exhibit potential immunosuppressive effects [Figure 2] in the TME and tumor-draining lymph nodes (TDLNs), which can enhance the likelihood of distant metastasis. The level of IDO1 in MDSCs is largely influenced by the STAT3-NF-κβ-IDO1 pathway. IDO1 is essential for recruiting MDSCs to lymph nodes, spleen, and tumor tissues for regional immunosuppression. Moreover, IDO1-producing MDSCs promote resistance to immunotherapy in several types of tumors, including BC.^48^ MDSCs expressing IDO1 have been found to accumulate and significantly suppress T cell activity while inducing Treg expression *in vitro*. The upregulation of IDO1 in MDSCs causes an increase in the infiltration of CD4+CD25+FoxP3+ Tregs, and alters the immunosuppressive function in breast neoplasms. Notably, IDO1-stimulated Tregs are involved in a feedback mechanism of recruiting and activating MDSCs.^49^ The ability of MDSCs to relocate to breast neoplasms in the absence of Tregs remains unclear.

Depletion of IDO1 expression reduced the ability of MDSCs to influence FoxP3+ Treg synthesis.^50^ IDO1 is directly associated with the development of tolerogenic DCs (tDCs) and their role in immunosuppression. Immunogenic DCs (iDCs) eventually transform into tDCs + IDO1 after activation by IFN-γ and lose their capacity to stimulate CD8+ T cells.^51^ The Wnt-β-catenin signaling cascade also promotes DC + IDO1 tolerance alongside IFN-γ.^52^ β-catenin, induced by the Wnt3a and Wnt5a receptors on DCs, attaches to the IDO1 promoter and increases IDO1 expression above that mediated by IFN-γ. IDO1 expression is primarily induced and maintained by Wnt5a, whereas IDO1 expression mediated by Wnt3a is dependent on IFN-γ.^53^ Tregs are stimulated by tDCs to differentiate, proliferate, and obstruct normal immune surveillance. IDO1 causes M2 TAMs to inhibit effector T cell responsiveness in the presence of IDO1-expressing BC cells. Additionally, AhR signaling induces M2 TAMs to express CD155, which mediates tumor immunosuppression.^54^ Continuous AhR signaling increases CD155 expression in TAMs. IDO1 indirectly regulates AhR expression in TAMs by recruiting the positive regulators STAT1 and STAT3.^55^ Through AhR signaling, TAMs work against anticancer immunity and tumor growth suppression. However, the dominant pathway in the TME remains to be clarified.

IDO1 regulates the anticancer effects of NK cells and their interaction with different TME-related cells. In some tumors, high levels of IDO1 and activation of AhR by Kyn through the JAK-STAT signaling cascade leads to a significant decrease in the levels of cytotoxic receptors, such as natural killer cell p46-related protein (NKp46) and natural killer cell group 2D (NKG2D) on the surface of NK cells.^56^ Some studies have reported that under high levels of the microRNA (miRNA) miR-18a, which significantly increases tumor cell development and metastasis and inhibits apoptosis in BC, IDO1 can dampen the expression of NKG2D and natural killer cell group 2D ligand (NKG2DL) in NK cells.^57^ The IDO1 pathway stimulates innate immune B cells to induce B regulatory cells (iBregs) that regulate T cells and promote the development of Foxp3+ Treg cells. This modification may contribute to the generation of an immunosuppressive regional milieu that favors tumor growth. Further research is necessary to understand the relationship between IDO1 and B cells.

## IDO1 and immunosuppression in breast cancer

**Figure 3:**
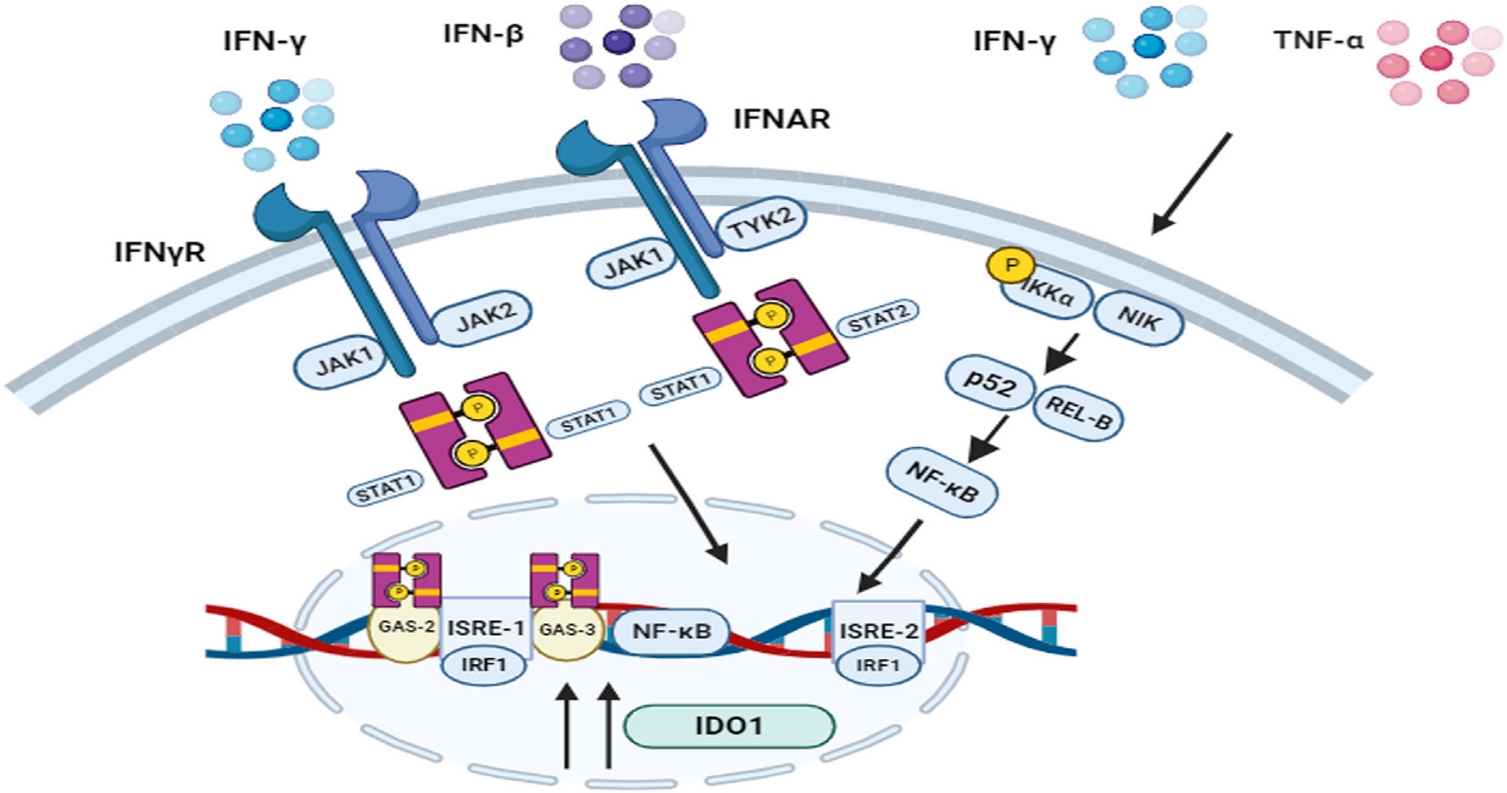
Activation and regulation of *IDO1* transcription by IFN-γ in BC. BC: Breast cancer; GAS-2: Growth arrest-specific 2; GAS-3: Growth arrest-specific 3; IDO1: Indoleamine 2,3-dioxygenase 1; IFNAR: Interferon-alpha/beta receptor; IFN-β: Interferon beta; IFN-γ: Interferon gamma; IFNγR: Interferon-gamma receptor; IKKα: Inhibitory-κB kinase α; IRF1: Interferon regulatory factor 1; ISRE: Interferon-stimulated response elements; JAK1: Janus kinase 1; JAK2: Janus kinase 2; NF-κB: Nuclear factor kappa B; NIK: Nuclear factor kappa B (NFκB)-inducing kinase; STAT 1: Signal transducer and activator of transcription 1; STAT 2: Signal transducer and activator of transcription 2; TNF-α: Tumor necrosis factor-α; TYK2: Tyrosine kinase 2.

IDO has been shown to suppress antitumor immunity and support metastasis, both of which are associated with BC development. The expression of IDO (or TDO, if secreted by cancer cells) by malignant cells within the tumor may promote the localized repression of the immune response. Many human cancers, including BC, express IDO1, and its upregulated expression has been linked to an unfavorable prognosis. In BC, IFN-γ is an efficient activator of IDO1, and its transcription-inducing mechanism relies on interferon regulatory factor 1 (IRF1), STAT1, and JAK [Figure 3].^58^ IFN-γ stimulates JAK to phosphorylate STAT1. The dimerized form of STAT1 then binds to the growth arrest-specific (GAS)-2 and −3 regions situated upstream of the *IDO1* coding region and triggers the transcription of *IDO1* and *IRF1*. Combined with STAT1, IRF1 binds to the interferon-stimulated response element (ISRE)-1 and ISRE-2 sites to stimulate the expression of IDO1. Interferon beta (IFN-β) boosts IDO1 levels by triggering JAK1/tyrosine kinase 2 (TyK2) and STAT1/STAT2 complex formation and stimulating the IDO1-Kyn-AhR signaling cascade, similar to IFN-γ.^59^ Alternatively, NF-κB-inducing kinase (NIK), activates inhibitory-κB kinase α (IKKα), which generates p52-Rel-B heterodimers.^60^ These heterodimers facilitate NF-κB translocation and bind to the coding region of *IDO1*. Other cytokines such as TNF-α and IL-6 can also stimulate IDO1 expression in BC cells. Through NF-κB translocation, TNF-α can synergistically stimulate the transcription of IDO1 and binding to GAS and ISRE, which are then transactivated by IFN-γ. The enzyme cyclooxygenase-2 (COX-2) controls the rate of prostaglandin synthesis and is strongly associated with the catalytic activity of IDO1.^61^ In BC, prostaglandin E2 stimulates the production of IDO1 by catalyzing protein kinase A (PKA) invasion and cAMP formation. The catalytic activity of IDO1 is subsequently stimulated by signals from the toll-like receptors (TLRs) or TNF receptors (TNF-Rs).

**Figure 4:**
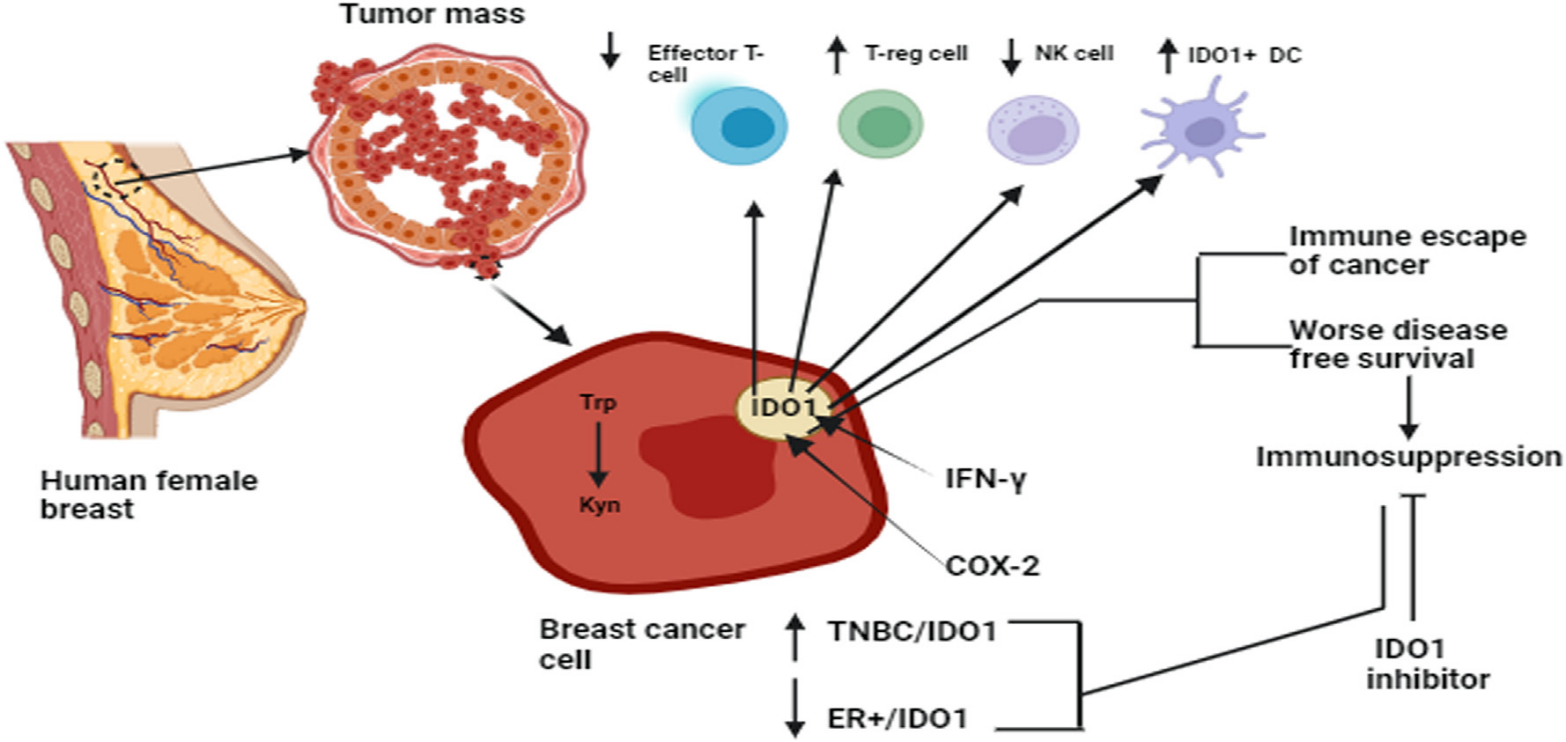
IDO1 promoting BC development by catalyzing the formation of Kyn from Trp causing a reduction in effector T cell and NK cells and an increase in the number of IDO1+ DC and Tregs. BC: Breast cancer; COX-2: Cyclooxygenase-2; ER+: Estrogen receptor positive; IDO1: Indoleamine 2,3-dioxygenase 1; IDO1+ DC: IDO1+ dendritic cell; IFN-γ: Interferon gamma; Kyn: Kynurenine; NK: Natural killer; TNBC: Triple-negative breast cancer; Treg: Regulatory T cell; Trp: Tryptophan.

The initial step to immune evasion involves the excessive activation and accumulation of IDO1 to quickly diminish Trp expression in the surrounding TME, which impedes the viability and proper functioning of effector T cells. IDO1 showed increased activity in cancerous cells and blood samples obtained from patients with BC. Elevated levels of IDO1 hampered T and NK cell-induced immunity and promoted the maturation of Tregs [Figure 4]. T cells experienced mid-G1 phase arrest due to the depletion of Trp from the TME, which is mediated by an increased concentration of tumorigenic IDO1. This is supported by evidence showing that IDO1 is highly expressed at metastatic locations and positively correlated with the immunosuppressive Treg population. In addition to the presence of Treg cells in the surrounding TME, an increase in MDSCs and IDO1+ tDCs has also been observed in the neoplasm and axillary lymph nodes of patients with BC.

The role of IDO1 in BC pathogenesis is summarized in Table 1. Yu et al. noted that the influx of Tregs into the TME by IDO1 activation in primary BC may suppress the local immune response and support metastasis.^62^ Song et al. proved that L-Kyn, an IDO1 catabolite, can induce apoptosis in NK cells by stimulating the release of ROS, thereby promoting immune evasion.^63^ In 2013, Yu et al. reported that the expression of IDO1 caused MDSCs to exert immunosuppressive effects in BC in a STAT3-dependent manner.^64^ In 2015, Puccetti et al. found that IDO1 expression was higher in BC than in colorectal cancer (CRC) based on the higher expression of Kyn in 18 and 14 out of 69 BC and CRC specimens, respectively.^65^ However, further comparisons with other types of cancers are needed to validate this observation.

**Table 1:** Important findings on IDO1 expression in patients with breast cancer.

Year	References	Findings
2011	Yu et al.^62^	Influx of T-regs into the TME through IDO1 activation in primary BC decreases the local immune response and supports metastasis.
2011	Song et al.^63^	L-Kyn induces apoptosis in NK cells by stimulating the release of reactive oxygen species.
2013	Yu et al.^64^	IDO1 causes MDSCs to exert immunosuppressive effects in BC in a STAT3-dependent manner.
2015	Puccetti et al.^65^	IDO1 expression is higher in BC than that in other cancers.
2017	Dewi et al.^66^	ER-α expression negatively correlates with IDO1 expression in ER + luminal-A and -B subtypes of BC.
2017	Li et al.^67^	Simultaneous expression of IDO1 and IL-6 is associated with advanced breast cancer and poor response to neoadjuvant chemotherapy.
2018	Wei et al.^68^	IDO1 increased the rate of angiogenesis in BC.
2018	Dill et al.^69^	IDO1 expression is highest in TNBC cases and 70% of PD-L1+ breast neoplasms express IDO1.
2019	Asghar et al.^70^	High COX-2 expression was associated with elevated IDO1 expression in patients with BC.
2020	Wei et al.^71^	Combination of IDO1 with tumor-infiltrating immune cells could be useful for determining prognosis in BC.
2020	Costa et al.^72^	IDO1 is expressed in 40% of HER2-expressing BC; a synergistic approach of treatment may be beneficial.
2021	Wei et al.^73^	5-HTP coheres to the promoter of IDO1 and upregulates its transcription.

5-HTP: 5-Hydroxytryptophan; BC: Breast cancer; COX-2: Cyclooxygenase-2; ER-α: Estrogen receptor-α; ER+: Estrogen receptor positive; HER2: Human epidermal growth factor receptor 2; IDO1: Indoleamine 2,3-dioxygenase 1; IL-6: Interleukin-6; L-kyn: l-Kynurenine; MDSC: Myeloid derived suppressor cell; NK: Natural killer; PD-L1: Programmed death ligand 1; STAT 3: Signal transducer and activator of transcription 3; TME: Tumor microenvironment; TNBC: Triple-negative breast cancer.

Epigenetics, especially DNA methylation patterns, play an important role in the expression of IDO1; for example, methylation of the *IDO1* promoter significantly reduces the expression of IDO1. Dewi et al. found that estrogen receptor-α (ER-α) expression was negatively correlated with IDO1 expression in ER + luminal-A and -B subtypes of breast tumors.^66^ Compared to patients with ER– BC, serum Kyn and IDO1 levels were markedly reduced in patients with ER + BC, and *ER* messenger ribonucleic acid (mRNA) levels were inversely correlated with the expression of IDO1. The IDO1 promoter was hypermethylated in ER + relative to ER– breast tissues, based on 450k, MassARRAY, pyrosequencing, and whole genome bisulfite sequencing. Additionally, the ER + BC cell lines showed a remarkable reduction in IDO1 expression. This poor expression of IDO1 caused by hypermethylation of the *IDO1* promoter with overexpression of ER suggests that inhibitors of IDO1 may represent a viable treatment against ER-related breast malignancies in combination with other therapeutic methods, specifically because the upregulation of IDO1 levels corresponds with both the aggressive phenotype and poor prognosis of ER-related breast malignancies.

Li et al. investigated the simultaneous expression of IL-6 and IDO1 in patients with BC before neoadjuvant chemotherapy; they identified an association between IL-6 and IDO1 expression in advanced BC and an inferior response to neoadjuvant therapy.^67^ Wei et al. showed that the expression of IDO1 was positively correlated with that of CD105 in Michigan Cancer Foundation (MCF)-7 malignant BC cell lines. This was associated with the initial metastasis of TDLNs, tumor lymph node metastasis (TNM) stage, and histological grade.^68^ In addition to suppressing immune responses, IDO1 can potentially contribute to tumor progression by enhancing the rate of angiogenesis by causing the proliferation of CD105-expressing human umbilical vein endothelial cells.^68^ Dill et al. showed that IDO1 expression was highest in TNBC, and approximately 70% of PD-L1+ breast neoplasms expressed IDO1. Most breast neoplasms with low PD-L1 expression were negative for IDO1.^69^ Ashgar et al. reported that high COX-2 expression was associated with elevated IDO1 expression in BC.^70^ In 2020, Wei et al. suggested that IDO1 and tumor-infiltrating immune cells are important prognostic markers in BC.^71^ Costa et al. suggested that 40% of HER2+ breast tumors overexpressed IDO1, indicating the potential for a synergistic treatment approach similar to that in TNBC models.^72^

In 2021, Wei et al. concluded that the increased expression of guanosine triphosphate (GTP) cyclohydrolase 1 (GCH1) was associated with lower average cell viability in TNBC and increased infiltration of Treg cells.^73^ GCH1 boosted Treg infiltration, decreased apoptosis, and increased the proportion of programmed cell death protein 1 (PD-1)-positive cells both *in vitro* and *in vivo*. A metabolomics study showed that Trp metabolism was reprogrammed by GCH1 overexpression, causing the accumulation of L-5-hydroxytryptophan (L-5-HTP) in the cytoplasm, and an increase in Kyn and a decrease in Trp expression in the supernatant. AhR activated by 5-HTP binds to the promoter of IDO1 to upregulate its transcription.^73^ Additionally, 2,4-diamino-6-hydroxypyrimidine (DAHP)-mediated suppression of GCH1 lowered IDO1 expression, inhibited tumor growth, and improved tumor response to PD-1 blocking immunotherapy.^73^

## Current status of clinical trials and future perspectives

Based on the role of IDO1 in facilitating immune escape and its abundant expression in the majority of tumor tissues, the performance of 11 inhibitors of IDO1 signaling has been investigated in clinical trials. IDO1 as a standalone therapy, a combination of IDO1 with chemotherapy and radiation, and a combination of IDO1 and checkpoint inhibitors have emerged in some recent clinical trials. Although various standalone IDO1 inhibitors, such as epacadostat and indoximod, have advanced to phase II clinical investigations, no discernible improvements in T cell count or tumor size were observed in preclinical trials.^74^^,^^75^ IDO1 is co-expressed with the immunological checkpoint PD-1/PD-L1 in various tumors,^76^^,^^77^ including BC, and anti-cytotoxic T-lymphocyte–associated antigen (CTLA)-4 and anti-PD-1/PD-L1 therapies increase IDO1 expression, indicating a potentially beneficial interaction between the three molecules.^78^ Epacadostat and the PD-1 antibody pembrolizumab together demonstrated promising treatment outcomes and advanced to phase III clinical studies,^41^ but showed no further improvements in clinical outcomes.^79^ The triple therapy combination comprising anti-CTLA-4, anti-PD-1/PD-L1, and an IDO1 inhibitor significantly reduced Treg cell infiltration *in vivo*. However, it showed no notable difference in terms of viability compared with PD-L1/CTLA-4 dual therapy. A recent study on 32 tumors showed that, by upregulating Kyn production, IL4I1 showed a stronger positive correlation with AhR activity than IDO1 expression.^80^ This may elucidate the shortcomings of IDO1 inhibitors in clinical trials. When IDO1 expression is downregulated, TDO and IDO2 may act as alternative pathways that hinder the functioning of IDO1 inhibitors. Therefore, future studies should consider a combination therapy that blocks the actions of TDO and IDO. Table 2 provides a summary of IDO1 inhibitors in current clinical trials.

**Table 2:** Ongoing clinical trials of IDO1 inhibitors.

Agent	Indications	Identifier	Phase	Strategy	Status	Efficacy
Indoximod (1-D-MT)	Metastatic breast cancer Metastatic glioma Metastatic prostate cancer Melanoma Metastatic pancreatic cancer	NCT01792050 NCT01042535 NCT02052648 NCT01560923 NCT02073123 NCT02077881	II I/II I/II II I/II I/II	Combined with taxane chemotherapy Combined with adenovirus -p53 transduced dendritic cell vaccine Combined with radiation therapy/bevacizumab Combined with sipuleucel-T Combined with Ipilimumab Combined with gemcitabine and nab-paclitaxel	Discontinued Active, not recruiting Recruiting Recruiting Recruiting Recruiting	No major changes were recorded. Unknown Unknown SD = 50% Unknown Unknown
Epacadostat (INCB024360)	Metastatic breast cancer Gastrointestinal stromal tumors Melanoma Fallopian tube cancer, ovarian cancer	NCT02178722 NCT03291054 NCT02752074 NCT02118285	III I/II III I	Combined with pembrolizumab Single agent Combined with pembrolizumab Combined with fludarabine and cyclophosphamide	Active, not recruiting Recruiting Completed Recruiting	No significant changes observed. Unknown No significant differences were found between treatment groups. Unknown
Navoximod (GDC-0919)	Metastatic breast cancer	NCT02048709 NCT02471846	I I	Single agent Combined with atezolizumab	Recruiting Recruiting	No significant differences were found between treatment groups. No significant improvements were recorded compared to atezolizumab therapy alone.

p53: Tumor protein p53; SD: Stable disease.

## Discussion

Much attention has been paid to the potential roles of IDO1 in tumor immunobiology since the discovery that IDO1 modulates maternal–fetal tolerance. Trp catabolism promotes cancer progression by facilitating cancer-induced immune suppression and evasion. Substantial evidence over the past few years indicates that the upregulation of IDO1 in several malignancies causes Trp deprivation in the regional milieu, in turn suppressing the T cell-driven immune response. IDO1 expression and clinical outcomes have shown strong associations in a variety of cancers, including BC.^81^ However, the role of IDO1 in many carcinomas remains controversial because of inconsistent results. In CRC and endometrial, ovarian, and esophageal carcinomas, the presence of IDO1 predicts poor clinical prognosis.^81^ In contrast, patients with hepatocellular carcinoma and high levels of IDO1 exhibit a good prognosis.^81^ Although the majority of experimental studies show a positive association between IDO1 activity and breast tumor progression, one study found the opposite^82^ and another found no appreciable variations in the expression of IDO1 between tumorigenic and non-tumorigenic tissues.^83^ The same research group observed conflicting results in the activation of IDO1 in patients with invasive BC.^84^ While later studies revealed that IDO1 activity was remarkably reduced in patients with a greater number of metastatic abrasions than in patients with no or fewer metastatic abrasions,^84^ earlier studies indicated that IDO1 expression was positively correlated with the number of metastatic abrasions.^85^ The significant enhancement of the KP in TNBC and HER2-enriched BCs, but not in luminal BCs, may be related to the distribution of different BC subtypes in their samples. Usually, IFN-γ would boost the upregulation of IDO1 and support BC growth, but basal-like BC (BLBC) presents IFN-γ-induced IDO1-promoted restriction of breast neoplasm progression, raising several questions on the role of IFN-γ.^86^^,^^87^ Clinical evidence supports the importance of IDO1 activation in BC and the promising implications of IDO1 inhibitors in addition to conventional chemotherapy. It appears that more metastatic invasive variants of BC are linked to KP activation. Therefore, IDO1 could be a useful biomarker for differentiating malignant and benign BC tissues and developing relevant diagnostic, prognostic, and treatment technologies.

Future research on the inhibition of IDO1 is needed to address several key issues. First, we should determine whether IDO1 inhibition activates compensatory mechanisms. Because TDO and IDO2 are in identical nodes of the KP, inhibition of IDO1 may result in a feedback process that causes the overexpression of IDO2 and/or TDO. TDO can substitute IDO1 as a driver for KP during metastasis and was continuously expressed in a BC metastasis mouse model.^88^ Therefore, it can act as an enzyme substitute in the absence of IDO1. Considering that IDO2 and TDO are poorly known in the field of cancer, future studies should also consider whether blocking TDO and/or IDO2 could provide general efficacy and reduce the inherent or acquired resistance to IDO1 blockade.

## Conclusion

The success of ICI-based therapies is limited to a few patients with cancer. AA-catabolizing enzymes are involved in the inhibition of immune responses against several tumors. BC is a highly non-immunogenic condition comprising many subtypes. The diverse components of the immunosuppressive TME mediate the interaction between breast neoplasm cells and the regional microenvironment, thereby supporting cancer growth. IDO1 is a Trp dioxygenase that catalyzes the synthesis of Kyn from Trp and is a pivotal enzyme in the KP. It can produce an inflammatory surrounding milieu through its role as a central interface between inflammatory cytokines. The deprivation of Trp in the regional milieu inhibits the activities of immunogenic cells and promotes the functions of tolerogenic cells owing to excessive IDO1 levels. In BC, IFN-γ is regarded as a potent stimulator of IDO1, and the mechanism of *IDO1* transcription depends on IRF1, STAT1, and JAK. In addition to IFN-γ, the cytokines TNF-α, IFN-β, and IL-6 can also promote IDO1 expression in BC cells. COX-2 efficiently induces IDO1 by controlling the rate of synthesis of prostaglandin E2.

This review highlights several important associations between IDO1 expression and BC pathophysiology. Various inhibitors of IDO1 are undergoing clinical trials; however, no significant treatment responses have been recorded. Although the activities of TDO and IDO2 require more research, they can act as alternative pathways, rendering IDO1 inhibitors ineffective. Although most studies have established a direct relationship between elevated IDO1 levels and BC progression, some inconsistencies have been reported, raising questions on the role of IDO1 in cancer progression. Despite these inconsistencies, IDO1 is regarded as the key rate-limiting enzyme controlling the synthesis of KP metabolites and is frequently overexpressed in various malignancies. An extensive understanding of the KP may help to develop effective IDO1 inhibitors, track the treatment responses of patients with BC to adaptively adjust disease management, and interpret inconsistent findings with greater confidence.

## Funding

This research did not receive any specific grant from funding agencies in the public, commercial, or not-for-profit sectors.

## Author contributions

The author claims sole responsibility for the contents and conclusions of the review.

## Ethics statement

None.

## Data availability statement

The datasets used in this study are available from the corresponding author upon reasonable request.

## Conflict of interest

The authors declare that they have no known competing financial interests or personal relationships that could have appeared to influence the work reported in this paper.
